# The Heterogeneous Nuclear Ribonucleoprotein K (*hnrnpk*) Gene Targeted by miR-460a-5p Functions in the Gonadal Differentiation and Development in Chinese Tongue Sole (*Cynoglossus semilaevis*)

**DOI:** 10.3390/ani16091327

**Published:** 2026-04-27

**Authors:** Kaimin Li, Haipeng Yan, Qi Liu, Wenjie Li, Chengbin Gao, Songlin Chen

**Affiliations:** 1 State Key Laboratory of Mariculture Biobreeding and Sustainable Goods, Laboratory for Marine Fisheries Science and Food Production Processes, Qingdao Marine Science and Technology Center, Yellow Sea Fisheries Research Institute, Chinese Academy of Fishery Sciences, Qingdao 266071, China; li_kaimin2025@163.com (K.L.); yanhaipeng265438@gmail.com (H.Y.); qiliu_0049@163.com (Q.L.); l3174190185@163.com (W.L.); 2 Laboratory for Marine Fisheries Science and Food Production Processes, Qingdao Marine Science and Technology Center, Qingdao 266237, China

**Keywords:** *hnrnpk*, sex control, *Cynoglossus semilaevis*, regulatory mechanism, overexpression

## Abstract

Chinese tongue sole (*Cynoglossus semilaevis*), an economically important mariculture species in China, exhibits pronounced sexual dimorphism in growth, underscoring the importance of elucidating sex regulatory mechanisms for aquaculture development. Heterogeneous nuclear ribonucleoprotein K (*hnrnpk*) critically regulates mammalian reproductive development, yet its role in fish sex regulation remains elusive. Here, we systematically investigated the underlying function and mechanisms of *hnrnpk* in *C. semilaevis* through integrated molecular cloning, expression profiling, upstream regulatory analysis, functional assays, and transcriptome sequencing. We found that *hnrnpk* was highly expressed in the gonad and liver, with female-biased expression during gonadal development. Promoter activity assays revealed that *sox2* and *c-Jun* enhanced *hnrnpk* transcription, whereas *foxl2* and *ar* suppressed it. Additionally, *hnrnpk* was directly targeted by miR-460a-5p in *C. semilaevis*, revealing multi-level transcriptional and post-transcriptional regulation. Functional analyses showed that *hnrnpk* regulated *cyp19a1a* in a cell type-dependent and dose-sensitive manner: the expression of *cyp19a1a* was both upregulated in *hnrnpk*-knockdown ovarian cells and *hnrnpk*-overexpression testicular cells. Interestingly, *foxl2* was upregulated in *hnrnpk*-knockdown ovarian cells but suppressed in *hnrnpk*-overexpression testicular cells, which showed the distinct regulation mechanisms in the different sexual programs. Transcriptomic analyses further revealed that several sex-related genes (*sox9a* with downregulation, etc.) were significantly regulated, and cell development and cycle pathways were dramatically enriched in functional enrichment analyses. This might indicate that *hnrnpk* overexpression drives *C. semilaevis* testis (CSTE) toward feminization reprogramming through *sox9* switching and multi-pathway perturbations. Overall, our findings might reveal that *hnrnpk*, a female-biased gene regulated by miR-460a-5p and transcription factors, influences sex-related gene expression through *sox9* switching. This study will offer new insights for *C. semilaevis hnrnpk* into sex determination and also provide a potential target for monosex breeding in aquaculture.

## Simple Summary

Chinese tongue sole (*Cynoglossus semilaevis*) is an economically important fish species in China, but its aquaculture productivity is seriously constrained by sex-related issues. Specifically, females grow much faster and larger than males, yet some genetic females naturally change sex into pseudomales under farming conditions, reducing the number of females in the population. Therefore, understanding how sex is regulated in this species is crucial for developing techniques to produce all-female stocks. In this study, we investigated a gene called *hnrnpk* and its role in sex regulation. We found that *hnrnpk* is more active in females than in males and is controlled by several upstream regulators, including transcription factors and a microRNA (miR-460a-5p). Experiments showed that *hnrnpk* can influence the expression of other sex-related genes in a cell type-dependent manner. Additionally, when we artificially increased *hnrnpk* expression in testicular cells, we observed changes in gene-splicing patterns and cell-signaling pathways. Together, these findings identify *hnrnpk* as an important player in gonadal development and suggest it could serve as a potential target for sex control strategies to improve aquaculture efficiency.

## 1. Introduction

Chinese tongue sole (*Cynoglossus semilaevis*) is an economically important mariculture species in China that exhibits pronounced sexual dimorphism and characterizes the faster growth and larger body sizes in females than males. Meanwhile, *C. semilaevis* possesses a ZW/ZZ genetic sex determination system, except genetic factors. Sex differentiation is also susceptible to temperature and other environmental factors. In an aquaculture environment, some genetic females (ZW) undergo sex reversal into physiological males (pseudomales), increasing male proportions in farmed populations and severely constraining aquaculture productivity [1,2]. This naturally occurring sex reversal reduces the availability of females that grow faster and larger than males. Consequently, all-female breeding stock is particularly precious. However, different fish species show diverse mechanisms for gender determination, and the detailed molecular regulation mechanisms of sex differentiation are still limited. Elucidating the molecular mechanisms underlying sex determination and differentiation in *C. semilaevis,* therefore, holds both theoretical significance and practical value for developing sex control technologies to enhance aquaculture efficiency.

Heterogeneous nuclear ribonucleoprotein K (*hnrnpk*), a crucial member of the HNRNP family, contains an N-terminal ROKNT domain and three tandem KH domains (KH1, KH2, KH3). KH domains function in RNA/DNA binding and protein–protein interaction, which enable *hnrnpk* to participate in transcriptional regulation, RNA splicing, translational control, DNA repair, and chromatin remodeling [3,4]. In teleost, *hnrnp* family genes, including *hnrnpk*, played multifaceted roles in development, immune regulation, and environmental stress response. For example, in zebrafish (*Danio rerio*), *hnrnpk* was expressed in multiple tissues during development, including the central nervous system, gut, otic vesicle, pectoral fin, and ventral mesoderm, and was predicted to participate in mRNA splicing and transcription regulation via RNA polymerase II [5]. In common carp (*Cyprinus carpio*), the hnrnp family member hnrnp A/B was shown to act as a negative regulator of type I interferon response, promoting viral replication by interacting with *mita* (mediator of IRF3 activation), *tbk1* (TANK-binding kinase 1), and *irf3* (interferon regulatory factor 3) to initiate their autophagic degradation [6]. In rainbow trout (*Oncorhynchus mykiss*), salinity stress induced alternative splicing of multiple *hnrnp* family members, including *hnrnpa0* (heterogeneous nuclear ribonucleoprotein A0), *hnrnp1a* (heterogeneous nuclear ribonucleoprotein 1A), *hnrnp1b* (heterogeneous nuclear ribonucleoprotein 1B), and *hnrnpc* (heterogeneous nuclear ribonucleoprotein C), which might affect RNA recognition motif (RRM) domain integrity and subsequently influence downstream target gene expression [7]. Although it has been extensively known that *hnrnpk* functions in viral infection and tumorigenesis, it also plays the key roles in reproduction, centering on precise post-transcriptional regulation, including mRNA stability, translation efficiency, and alternative splicing [8,9]. In mammals, *hnrnpk* is a key regulator of both male and female reproduction. In males, it plays essential roles in spermatogenesis; in females, it is involved in primordial follicle assembly and oocyte survival. For example, spermatogenic arrest and male infertility were induced in mouse spermatogonia by deleting *hnrnpk* [10]. It was also reported that primordial follicle assembly and development were disrupted in rat ovaries through knocking down the *hnrnpk*, thereby increasing oocyte apoptosis [11]. These studies adequately establish *hnrnpk* as an important regulator in mammalian reproductive development. However, few studies were reported on the function of *hnrnpk* in regulating sex-related gene expression in teleosts, let alone research on sex regulation. Therefore, it is urgent to explore the regulatory roles of *hnrnpk* in sex determination and differentiation in *C. semilaevis.*

In addition, the crucial roles of miRNAs in sexual regulation and differentiation systems have been much accounted for and reported in vertebrates. In mammals, the miR-17-92 cluster was essential for ovarian development, with its deletion leading to complete male-to-female sex reversal in XY mice [12]. In fish, multiple miRNAs were identified as key regulators of gonadal development, among which miR-26a-5p directly targeted *cyp19a1a* to facilitate female sex reversal in orange-spotted grouper (*Epinephelus coioides*) [13]. In common carp (*Cyprinus carpio*), miR-153b-3p regulated the proliferation and differentiation of male germ cells by targeting *amh* [14]. Notably, in *C. semilaevis*, ssa-mir-196a-4 was shown to directly target *lgr8* and play an important role in testis development [15]. These findings highlighted the conserved and critical functions of miRNAs in sex differentiation across teleosts. Meanwhile, miR-460 family members were reported to participate in vertebrate developmental regulation [16], while their regulation mechanisms for the expression of the downstream gene expressions in fish remain unclear. Also, the detailed regulation of miR-460-5p targeting *hnrnpk* to be involved in the sex control and differentiation in *C. semilaevis* still needs more efforts to explore.

Despite the growing understanding of *hnrnpk* functions in mammalian reproduction, its role in sex regulation and gonadal development in teleosts remains completely unknown. In particular, it is unclear whether *hnrnpk* is regulated by miRNAs and how it participates in sex-related gene expression and sex differentiation in fish. Here, we systematically investigated the expression profile, upstream regulatory mechanisms, and functional role of *hnrnpk* in sex-related gene expression and gonadal development in *C. semilaevis*. Our findings will provide new insights into RNA-binding protein mechanisms within sex determination networks and offer a potential molecular target for sex control in aquaculture.

## 2. Materials and Methods

### 2.1. Ethics Statement and Animal Euthanasia

All experimental procedures involving *C. semilaevis* were conducted in accordance with the guidelines established by the Experimental Animal Care, Ethics, and Safety Inspection Committee of the Yellow Sea Fisheries Research Institute, Chinese Academy of Fishery Sciences (Approval No.: YSFRI-2025019). Fish were anesthetized with 120 mg/L of MS-222 (Sigma, Darmstadt, Germany) to minimize suffering prior to euthanasia.

### 2.2. Cell Culture

Human embryonic kidney (HEK 293T) cells were cultured in high-glucose DMEM medium (Solarbio, Beijing, China) supplemented with 10% fetal bovine serum (FBS; Gibco, Grand Island, NY, USA) and 1% penicillin/streptomycin/amphotericin antibiotic solution (Solarbio, Beijing, China). Cells were cultured in an ESCO CO_2_ incubator (ESCO Micro Pte Ltd., Singapore) at 37 °C in a humidified atmosphere containing 5% CO_2_.

*C. semilaevis* testis (CSTE) [17] and ovary (CSO) [18] cells were cultured in L-15 medium (Solarbio, Beijing, China) supplemented with 20% FBS, 1% penicillin/streptomycin/amphotericin antibiotic solution, 14 mM of β-mercaptoethanol (VWR, Radnor, PA, USA), 5 ng/mL of epidermal growth factor (EGF; Beyotime, Shanghai, China), 5 ng/mL of basic fibroblast growth factor (bFGF; Beyotime, Shanghai, China), and 5 ng/mL of leukemia inhibitory factor (LIF; Beyotime, Shanghai, China). These cells were maintained at 24 °C in SANYO incubator (SANYO Electric Co., Ltd., Osaka, Japan) for subsequent experiments.

### 2.3. Fish Samples, Genetic Sex Identification, Total RNA Extraction, and cDNA Synthesis

The experimental fish were obtained from Weizhuo Aquatic Technology Company (Tangshan, Hebei, China). Genetic sex identification was performed following our previously established methods [19]. Male and female individuals were dissected at different developmental stages (40 days, 60 days, 3 months, 5 months, 8 months, 1 year, and 2 years), and tissues, including liver, spleen, kidney, intestine, gill, brain, skin, and gonad, were collected. Due to the small size of individuals at 40 and 60 days post-hatching, whole visceral masses were collected instead of individual tissues. Moreover, gonad tissue and the surrounding muscle tissue were inevitably collected and were regarded as the gonadal samples. Three biological replicates of sampled tissues were designed for each time point of developmental stage. All tissue samples were immediately preserved in liquid nitrogen.

Total RNA was extracted using TRIzol reagent (Invitrogen, Carlsbad, CA, USA) following the manufacturer’s instructions. RNA integrity was assessed by 0.8% agarose gel electrophoresis (visualization of intact 28S and 18S ribosomal RNA bands), and concentration and purity were determined using a NanoDrop spectrophotometer (Thermo Fisher Scientific, Waltham, MA, USA) to confirm all extracted samples with an A260/280 ratio > 1.8. The first strand of cDNA was synthesized using the PrimeScript RT Reagent Kit with gDNA Eraser (TaKaRa, Kusatsu, Japan) according to the manufacturer’s instructions and stored at −80 °C.

### 2.4. Molecular Cloning, Phylogenetic Analysis, and Structural Characterization of the Hnrnpk Coding Region

The *hnrnpk* sequence was obtained from the genome and transcriptome databases of *C. semilaevis* established by our laboratory [1,20]. Primers for amplifying the coding sequence (CDS) of *hnrnpk* were designed using Primer Premier 5.0 (Table 1). Full-length cDNA was synthesized using the First Strand cDNA Synthesis Kit ReverTra Ace-α (Toyobo, Osaka, Japan). The target fragments were amplified and cloned into the 5 min TA/Blunt-Zero Cloning Kit (Vazyme, Nanjing, China). The recombinant plasmids were subsequently transformed into DH5α Chemically Competent Cells (Coolaber, Beijing, China). Positive clones were selected and subjected to Sanger sequencing at Sangon Biotech (Shanghai, China).

Based on the CDS sequence of *hnrnpk*, homologous sequences from other vertebrates were retrieved via BLAST (https://blast.ncbi.nlm.nih.gov, accessed on 28 February 2026) search against the NCBI database [21]. Amino acid sequences of HNRNPK from 16 vertebrate species, including *Solea senegalensis*, *Paralichthys olivaceus*, *Epinephelus lanceolatus*, *Hippoglossus stenolepis*, *Hippoglossus hippoglossus*, *Platichthys flesus*, *Pleuronectes platessa*, *Xiphophorus maculatus*, *Limanda limanda*, *Larimichthys crocea*, *Scophthalmus maximus*, *Danio rerio*, *Homo sapiens*, *Mus musculus*, *Gallus gallus*, and *Lampetra fluviatilis*, were collected for sequence alignment and phylogenetic analysis. Phylogenetic analysis was performed using the maximum likelihood method. In detail, sequences were aligned with ClustalW implemented in MEGA12 [22], and the alignment results were visualized using the ESPript 3.2 online server (https://espript.ibcp.fr, accessed on 20 April 2026). The best-fitting substitution model was selected based on the Bayesian Information Criterion (BIC). The JTT+G model (Jones-Taylor-Thornton with gamma-distributed rate heterogeneity among sites) was selected as the optimal model for tree reconstruction. A maximum likelihood tree was reconstructed under this model with five discrete gamma categories, and the tree was rooted using *Lampetra fluviatilis* as an outgroup. Nodal support was assessed using 1000 bootstrap replicates [22]. All sites were used in the analysis without any filtering for gaps or missing data. For domain analysis, amino acid sequences of HNRNPK from 17 vertebrate species were submitted to NCBI Batch CD-Search against the CDD database with default parameters (E-value = 0.01) [23,24]. Identified domains were visualized using TBtools-II (v2.471) [25]. Additionally, the molecular weight and theoretical pI of the encoded protein were predicted using ExPASy (https://web.expasy.org/protparam/, accessed on 28 February 2026).

### 2.5. Expression Analysis of Hnrnpk in Different Tissues and Gonadal Developmental Stages of C. semilaevis

Specific primers for *hnrnpk* were designed using Primer Premier 5.0, with *β-actin* (Table 1) serving as the internal reference gene [26]. The expression patterns of *hnrnpk* were examined in various tissues (gonad, muscle, liver, kidney, brain, and intestine) from 5-month-old individuals, a stage characterized by rapid gonadal development and the emergence of sexual dimorphism, as well as in gonads at different developmental stages (40 days, 60 days, 3 months, 5 months, 8 months, 1 year, and 2 years). Three biological replicates were used for each tissue and each developmental stage. In brief, quantitative real-time PCR (qPCR) was performed using an ABI 7500 Fast real-time PCR system (Applied Biosystems, Foster City, CA, USA) with THUNDERBIRD^®^ SYBR^®^ qPCR Mix (Toyobo, Osaka, Japan) according to the manufacturer’s instructions. The cDNA synthesized with the PrimeScript RT Reagent Kit with gDNA Eraser (TaKaRa, Kusatsu, Japan) was used as template. The thermal cycling conditions were as follows: 95 °C for 30 s, followed by 40 cycles of 95 °C for 5 s and 60 °C for 34 s. Melting curve analysis was performed to confirm amplification specificity. Relative expression levels were calculated using the 2^−ΔΔCt^ method.

### 2.6. Cloning and Functional Analysis of the Hnrnpk Promoter

To investigate the transcriptional regulation mechanism of *hnrnpk*, the promoter region was first predicted. Based on the genomic sequence of *C. semilaevis*, the 2000 bp region upstream of the ATG start codon was subjected to bioinformatic analysis using the online database AnimalTFDB v4.0 (https://guolab.wchscu.cn/AnimalTFDB4//#/, accessed on 28 December 2025). A 1954 bp candidate promoter region was identified, which contained typical core promoter elements and multiple putative transcription factor binding sites, including *sox2*, *c-Jun*, *foxl2*, and *ar*. The putative promoter region of *hnrnpk* was amplified by PCR using specific primers. The purified PCR product was inserted into the *Hind*III-linearized pGL3-Basic vector (Promega, Madison, WI, USA) using the TOROIVD^®^ One Step Fusion Cloning MIX Seamless cloning kit (TOROIVD, Shanghai, China), generating the reporter plasmid pGL3-*hnrnpk*. For luciferase reporter assays, HEK293T cells were seeded in 24-well plates and transiently transfected with 800 ng/well of pGL3-*hnrnpk*, pGL3-Control, and pGL3-Basic using Lipo8000™ transfection reagent (Beyotime, Shanghai, China), respectively. To normalize transfection efficiency, 40 ng/well of the pRL-TK *Renilla* luciferase plasmid was co-transfected as an internal control. After 48 h of incubation, cells were lysed, and firefly and *Renilla* luciferase activities were then measured sequentially using the Dual-Luciferase Reporter Assay System (Beyotime, Shanghai, China) on a Varioskan Flash multimode microplate reader (Thermo Fisher Scientific, Vantaa, Finland). Relative luciferase activity was calculated by normalizing firefly luciferase activity to *Renilla* luciferase activity.

To investigate the regulatory effects of transcription factors on *hnrnpk* promoter activity, the previously constructed overexpression plasmids pcDNA3.1-*sox2*, pcDNA3.1-*c-Jun*, pcDNA3.1-*foxl2*, and pcDNA3.1-*ar* were used for subsequent experiments. These plasmids carried the coding sequences of *sox2*, *c-Jun*, *foxl2*, and *ar*, respectively, and were constructed using the pcDNA3.1 vector (Invitrogen, Carlsbad, CA, USA). Each TF expression construct was co-transfected into HEK293T cells with the pGL3-*hnrnpk* reporter plasmid, after which luciferase activity was measured as described above.

### 2.7. Dual-Luciferase Reporter Assay

To validate the targeting relationship between miR-460a-5p and *hnrnpk*, a dual-luciferase reporter system was employed. Potential miRNAs targeting *hnrnpk* were predicted using RNAhybrid software (v2.2.1), and miR-460a-5p was selected based on the optimal minimum free-energy value [27]. The 3′ untranslated region (UTR) fragment of *hnrnpk* mRNA was amplified by PCR and inserted into the pmirGLO vector (Promega, Madison, WI, USA) between the *Nhe*I and *Sal*I restriction sites. The resulting wild-type reporter plasmid (*hnrnpk*-3′UTR-WT) was confirmed by sequencing. Subsequently, a mutant reporter plasmid (*hnrnpk*-3′UTR-MUT) was generated by site-directed mutagenesis of the wild-type construct using mutation-specific primers and the Fast MultiSite Mutagenesis System (FM201-01; TransGen Biotech, Beijing, China), following the manufacturer’s instructions. miR-460a-5p mimics and negative control mimics were designed and synthesized. For luciferase reporter assays, HEK293T cells were co-transfected with either the wild-type or mutant reporter plasmid, along with either miR-460a-5p mimics or negative control mimics, using Lipo8000™ transfection reagent (Beyotime, Shanghai, China). This cell line has been widely used for miRNA target validation in fish species [28,29]. After 48 h of transfection, luciferase activities were measured as described in Section 2.5.

### 2.8. Knockdown and Overexpression

For the knockdown experiment, three small interfering RNAs (siRNAs) targeting the CDS of *hnrnpk*, labeled as siRNA-1, siRNA-2, and siRNA-3, were designed and synthesized by Sangon Biotech (Shanghai, China). The siRNA sequences and the primers for *hnrnpk*-interacting genes are listed in Table 1. For transfection, the CSO were seeded and transfected with each siRNA at a final concentration of 50 nmol/L using the riboFECT™ CP Transfection Kit (RiboBio, Guangzhou, China). A non-silencing siRNA (siRNA-NC) was used as a negative control. All transfections were performed in three biological replicates. At 72 h post-transfection, cells were harvested for the subsequent total RNA extraction. For the overexpression, Primers (Table 1) containing homology arms of the pcDNA3.1 vector were designed to amplify the coding sequence of *hnrnpk*. The purified PCR product was cloned into the pcDNA3.1 vector linearized with *Hind*III using the TOROIVD^®^ One-Step Fusion Cloning Kit, generating the recombinant plasmid pcDNA3.1-*hnrnpk*. CSTE cells seeded in six-well plates were transiently transfected with pcDNA3.1-*hnrnpk* or the empty vector (as a negative control) using Lipo8000™ Transfection Reagent according to the manufacturer’s instructions. At 48 h post-transfection, cells were harvested for the following experiments. Subsequently, the total RNA of the harvested cells above was extracted using TRIzol reagent. RNA extraction, cDNA synthesis, and qPCR were performed as described in Section 2.2. The knockdown efficiency of each siRNA was first verified by qPCR. The expression patterns of *hnrnpk* and other sex-related genes were determined and analyzed by qPCR.

### 2.9. RNA Extraction, Library Construction, and Sequencing for Hnrnpk-Overexpression CSTE Cells

For the transcriptome analysis, both the *hnrnpk*-overexpression group and the empty vector control group were set up with three biological replicates. Total RNA was isolated from CSTE cells overexpressing *hnrnpk* and those transfected with the empty vector (negative control) using TRIzol reagent (Thermo Fisher Scientific, Waltham, MA, USA) according to the manufacturer’s instructions. RNA quality was assessed using a Bioanalyzer 2100 (Agilent Technologies, Santa Clara, CA, USA), and samples with RNA integrity number (RIN) > 7.0 were used for library construction. mRNA was purified from 5 μg of total RNA using Dynabeads Oligo (dT) (Thermo Fisher Scientific, Waltham, MA, USA), fragmented, and reverse-transcribed into cDNA. The cDNA libraries were constructed using a strand-specific library preparation method with dual-index adapters, followed by PCR amplification (95 °C for 3 min; 8 cycles of 98 °C for 15 s, 60 °C for 15 s, and 72 °C for 30 s; final extension at 72 °C for 5 min) [30]. Library quality was validated, and paired-end sequencing (2 × 150 bp) was performed on the Illumina NovaSeq 6000 platform (San Diego, CA, USA). A total of 217 million clean reads were generated, with an average mapping rate of 97.1% to the reference genome. The raw sequencing data generated in this study were deposited in the NCBI Sequence Read Archive (SRA) under BioProject accession PRJNA1438903 and SRA study accession SRP684648.

### 2.10. Differential Expression Analyses

Raw reads were filtered to remove adapter-contaminated reads, polyA/polyG reads, and low-quality reads using Cutadapt [31]. Clean reads were aligned to the *C. semilaevis* reference genome established by our laboratory using HISAT2 (v2.2.1) [32]. Transcript assembly and quantification were performed using StringTie (v2.1.6) [30]. Differential expression analysis between the negative control group and the *hnrnpk* overexpression group was conducted using DESeq2, with significantly differentially expressed genes (DEGs) defined as |fold change| ≥ 2 and false discovery rate (FDR) < 0.05 [33].

Functional enrichment analysis of DEGs was performed using Gene Ontology (GO) and KEGG pathway databases, with *p* < 0.05 considered statistically significant. Gene set enrichment analysis (GSEA) was conducted using GSEA software (v4.1.0) to identify significantly enriched biological pathways. Alternative splicing events were analyzed using rMATS (v4.1.1), with FDR < 0.05 as the significance threshold. SNP/InDel calling and annotation were performed using Samtools (v0.1.19) and ANNOVAR (v2024Oct14) [34].

### 2.11. Validation of Differentially Expressed Genes by qPCR

To validate the reliability of the transcriptome data, 10 differentially expressed genes (DEGs) were selected for qPCR analysis, including *hnrnpk*, *pde4cl*, *suv39h1*, *lamb1l*, *ccne2l*, *sox9-A*, *sox9a*, *foxl2*, *cyp19a1a*, and *neurl3*. Total RNA was extracted from the same batches used for RNA-seq, and cDNA was synthesized as described in Section 2.3. The qPCR system was performed using specific primers (Table 1) under the same conditions described in Section 2.5. Relative expression levels were calculated using the 2^−ΔΔCt^ method with *β*-actin as the internal reference gene. All reactions were performed in triplicate biological repetition, and data were presented as mean ± SD.

### 2.12. Statistical Analysis for Data

Comparisons between two groups were performed using Student’s *t*-test, and comparisons among multiple groups were performed using one-way ANOVA. *p* < 0.05 was considered statistically significant, and *p* < 0.01 was considered extremely significant. All statistical analyses were performed using GraphPad Prism software (version 10.1.2, GraphPad Software, Boston, MA, USA).

## 3. Results

### 3.1. Molecular Characterization and Phylogenetic Analysis of Hnrnpk

The CDS of *hnrnpk* of *C. semilaevis* was cloned by PCR and determined to be 1302 bp length (Supplementary Figure S1), encoding a protein of 433 amino acids with a predicted molecular weight of 48.16 kDa and a theoretical isoelectric point of 6.74 (Table 2). High-sequence identities (ranging from 95.99% to 96.32%) were observed with other *hnrnpk* variants from the same species, further confirming the specificity of the cloned fragment.

**Figure 1:**
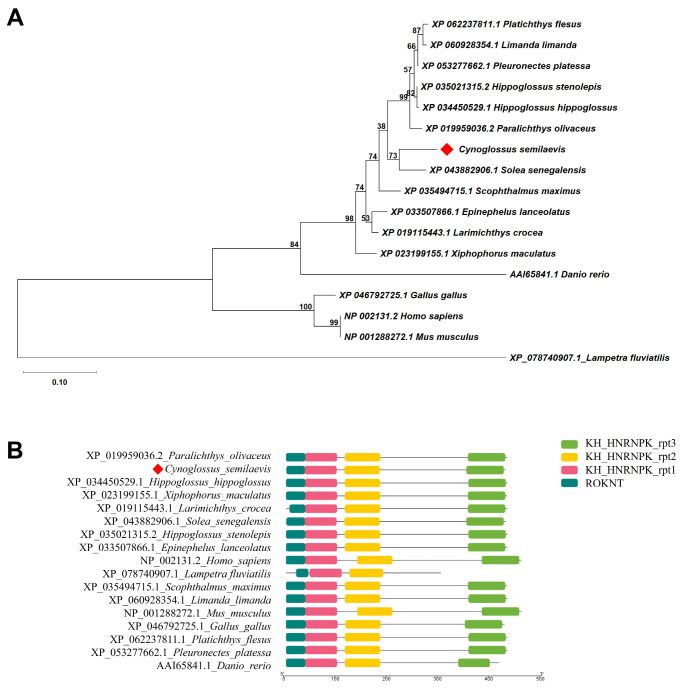
Phylogenetic tree and conserved domain analysis of HNRNPK. (**A**) Maximum likelihood phylogenetic tree constructed based on HNRNPK amino acid sequences from 17 vertebrate species. Numbers at nodes indicate bootstrap support percentages from 1000 replicates. The HNRNPK sequence of *C. semilaevis* is marked with a red diamond. (**B**) Schematic representation of conserved domains in HNRNPK proteins from 17 vertebrate species. The HNRNPK sequence of *C. semilaevis* is also marked with a red diamond. All sequences contain three canonical KH domains (KH_HNRNPK_rpt1, KH_HNRNPK_rpt2, and KH_HNRNPK_rpt3, indicated by red, yellow, and light green boxes, respectively) and a C-terminal ROKNT repeat region (indicated by a dark green box). Domain annotations were based on the NCBI CDD database, and visualization was generated using TBtools-II software.

To elucidate the evolutionary status of *C. semilaevis hnrnpk*, phylogenetic analysis and conserved domain prediction were performed on *hnrnpk* proteins from various vertebrate species. The resulting phylogenetic tree clearly clustered *hnrnpk* proteins according to their genetic relationships. The evolutionary status of *C. semilaevis hnrnpk* sequence was well supported by a monophyletic group with Pleuronectiformes species. Briefly, *hnrnpk* of *C. semilaevis* showed the closest relationship with *Solea senegalensis hnrnpk*, followed by *S. maximus, H. hippoglossus*, *H. stenolepis*, and *P. olivaceus* (Figure 1). Furthermore, conserved domain analysis showed that *hnrnpk* of *C. semilaevis* contained the characteristic three tandem KH domains (KH1, KH2, KH3) and a C-terminal ROKNT repeat region, which was similar to the structure of other teleost, as well as mammals, birds, and those higher vertebrates. These results revealed a high degree of structural conservation in *hnrnpk* proteins throughout the evolution from teleost fish to mammals (Figure 1 and Figure S2).

### 3.2. Expression Profiles of C. semilaevis hnrnpk During Gonadal Development and in Tissue Distribution

**Figure 2:**
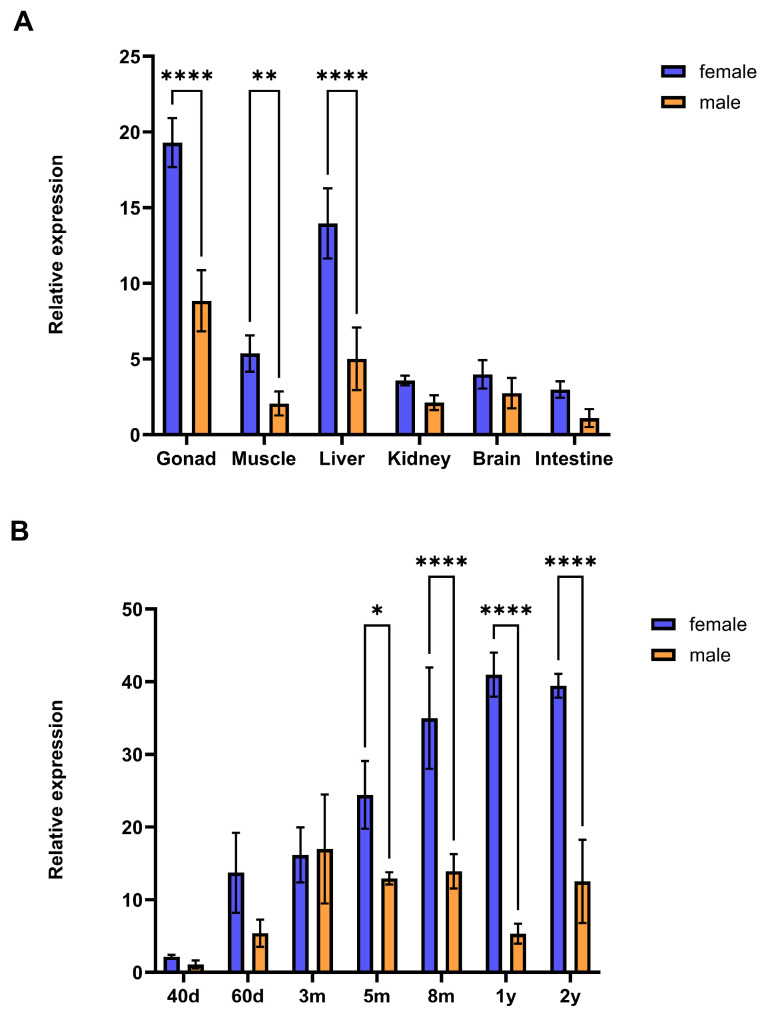
Expression patterns of *hnrnpk* in various tissues and during gonadal development of *C. semilaevis*. (**A**) Relative expression levels of *hnrnpk* in different tissues at 5m in *C. semilaevis* determined by qPCR. Examined tissues included gonad, muscle, liver, kidney, brain, and intestine. (**B**) Relative expression levels of *hnrnpk* in female and male gonads at different developmental stages: 40 days, 60 days, 3 months, 5 months, 8 months, 1 year, and 2 years. Data are expressed as mean ± SD (*n* = 3). * (*p* < 0.05), ** (*p* < 0.01), and **** (*p* < 0.0001) indicated significant differences between females and males.

To investigate the expression profiles of *C. semilaevis hnrnpk*, qPCR was performed to examine its temporal and spatial expression patterns. Tissue distribution analysis showed that *hnrnpk* was ubiquitously expressed in all examined tissues. The most predominant expression was found in the gonad, and followed by liver, muscle, brain, and so on. Notably, females exhibited consistently higher expression levels than males across all tissues, particularly in the gonad, liver, and muscle (Figure 2A). During gonadal development of *C. semilaevis*, *hnrnpk* displayed distinct sex-dependent expression patterns. In females, expression levels increased progressively with age, peaked at 1y. In the individual of 2y, the expression slightly decreased, and the expression profile still maintained a high level. In males, a rapid increasing expression was observed in the early developmental stages, which reached a peaked expression at 3m. At the other subsequent time points, the expressions were fluctuated at moderate levels. Similarly, a higher expression level was also found at each time point in females than that in males (Figure 2B).

### 3.3. Transcriptional Regulation of Hnrnpk: Promoter Activity Analysis and Transcription Factor Validation

**Figure 3:**
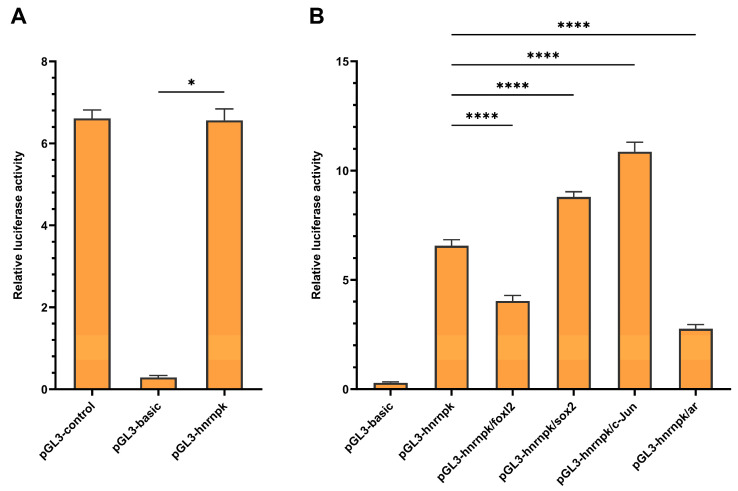
Promoter activity analysis and transcription factor validation of *hnrnpk*. (**A**) Dual-luciferase reporter assay of *hnrnpk* promoter activity. HEK293T cells were transfected with pGL3-*hnrnpk*, pGL3-Control (positive control), or pGL3-Basic (negative control), and luciferase activities were measured 48 h post-transfection. Data were presented as mean ± SD (*n* = 3). * (*p* < 0.05) indicated extremely significant differences compared to the negative control. (**B**) Regulatory effects of transcription factors (*sox2*, *c-Jun*, *foxl2,* and *ar*) on *hnrnpk* promoter activity. HEK293T cells were co-transfected with each transcription factor expression plasmid and pGL3-*hnrnpk*, and luciferase activities were measured 48 h post-transfection. Data were presented as mean ± SD (*n* = 3). **** (*p* <0.0001) indicates significant differences compared to the control group.

To investigate the transcriptional regulation of *hnrnpk*, a 1954 bp fragment identified from the predicted promoter region was cloned as the candidate promoter. Dual-luciferase reporter assays showed that this fragment exhibited significant promoter activity, with relative luciferase activity approximately 23-fold higher than that of the pGL3-Basic negative control (Figure 3A). Moreover, the functionality of the transcription factor binding sites was predicted. HEK293T cells were co-transfected with the pGL3-*hnrnpk* reporter plasmid and overexpression plasmids for *sox2*, *c-Jun*, *foxl2*, or *ar*. The results showed that *Sox2* and *c-Jun* significantly enhanced *hnrnpk* promoter activity (by approximately 1.34-fold and 1.65-fold, respectively), whereas *foxl2* and *ar* significantly suppressed it (by approximately 39% and 58%, respectively, Figure 3B).

### 3.4. Potential Regulatory Relationship Between Hnrnpk and miR-460a-5p

**Figure 4:**
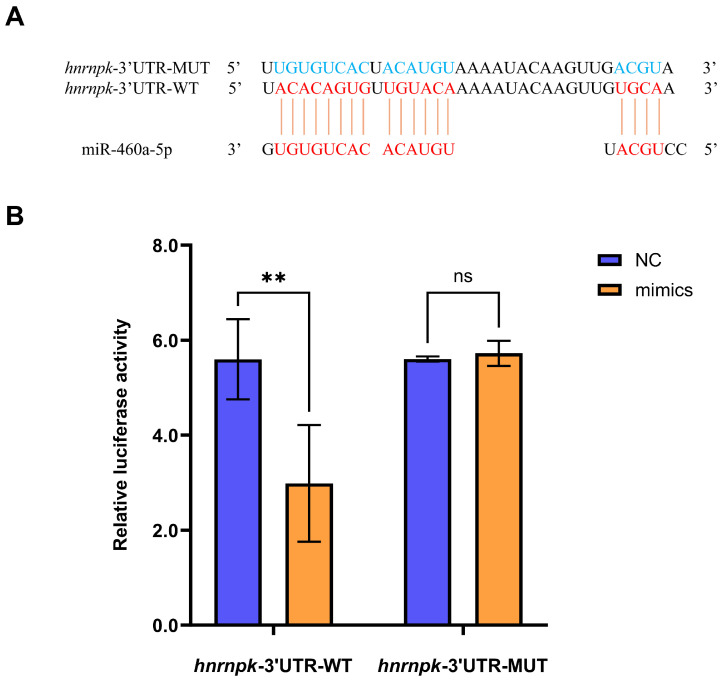
Targeting validation of *hnrnpk* by miR-460a-5p. (**A**) Schematic diagram of the miR-460a-5p binding site in the *hnrnpk* 3′UTR. The predicted seed region binding site for miR-460a-5p is indicated in red, and the mutated sequence is shown in blue. (**B**) Dual-luciferase reporter assay validating miR-460a-5p targeting of the *hnrnpk* 3′UTR. HEK293T cells were co-transfected with wild-type (WT) or mutant (MUT) 3′UTR reporter plasmids and miR-460a-5p mimics or negative control (NC), and luciferase activities were measured 48 h post-transfection. Data were mean ± SD (*n* = 3). ** (*p* < 0.01) indicated extremely significant differences compared to the NC group, ns, not significant.

Through the bioinformatic analysis, *hnrnpk* was predicted as a candidate target gene of miR-460a-5p, with putative binding sites identified in its 3′ untranslated region (UTR) (Figure 4A). To validate this targeting relationship, we conducted dual-luciferase reporter assays in HEK 293T cells. Co-transfection with the miR-460a-5p mimic significantly reduced the luciferase activity of the *hnrnpk*-3′UTR-WT reporter relative to the negative control. Conversely, mutation of the *hnrnpk*-3′UTR-MUT construct completely abrogated this inhibitory effect (Figure 4B). Meanwhile, the miRNA negative control group (NC) was also conducted to transfect with wild/mutant plasmids in the HEK 293T cells for luciferase activity, while no effect was observed on luciferase activity in these cells. These findings confirmed that *hnrnpk* could be a direct target of miR-460a-5p.

### 3.5. Cell Type-Dependent Regulation of Sex-Related Genes by Hnrnpk

**Figure 5:**
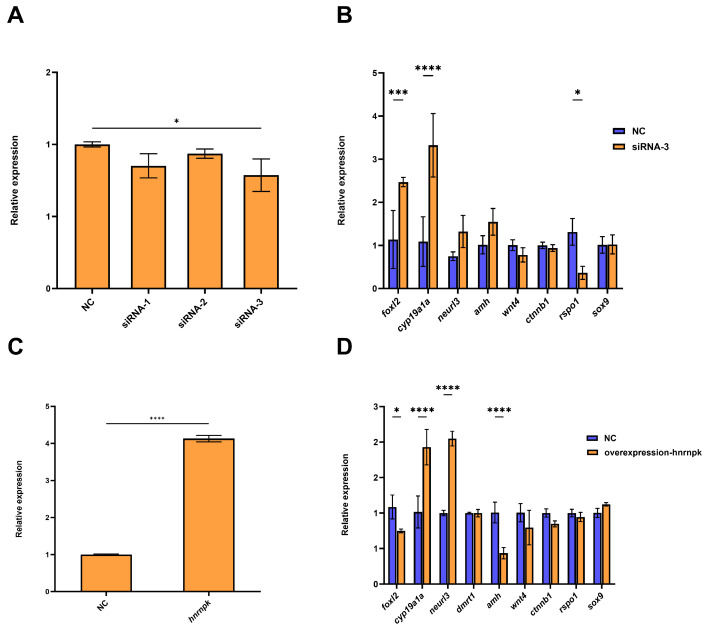
Cell type-dependent regulation of sex-related genes by *hnrnpk*. (**A**) Knockdown efficiency of three siRNAs in CSO cells. Cells were transfected with siRNA-1, siRNA-2, siRNA-3, or siRNA-NC (negative control), and *hnrnpk* expression levels were detected by qPCR. (**B**) Effects of *hnrnpk* knockdown on sex-related gene expression in ovarian cells. CSO cells were transfected with siRNA-3. (**C**) Overexpression efficiency of *hnrnpk* in CSTE cells. Cells were transfected with pcDNA3.1-*hnrnpk* or empty vector (NC), and *hnrnpk* expression levels were detected by qPCR. (**D**) Effects of *hnrnpk* overexpression on sex-related gene expression in CSTE. CSTE cells were transfected with pcDNA3.1-*hnrnpk* or NC. All data were mean ± SD (*n* = 3). * (*p* < 0.05), *** (*p* < 0.001), and **** (*p* < 0.0001) indicated significant differences compared to the NC group.

To investigate the role of *hnrnpk* in sex regulation, we performed loss-of-function and gain-of-function experiments in CSO and CSTE cells, respectively. For the knockdown experiment, three siRNAs were used to knock down *hnrnpk* in CSO, while siRNA-3 showed the lowest expression among these three siRNAs, which meant the strongest knockdown efficiency. Therefore, siRNA-3 was selected as the optimal candidate for the subsequent knockdown experiment (Figure 5A). In the *hnrnpk-*knockdown CSO cells, the expression of the female-related genes was affected, cytochrome P450 family 19 subfamily A member 1a (*cyp19a1a*) was significantly upregulated with the highest fold-change (3.1-fold change), and *foxl2* was also significantly upregulated with 2.2-fold change, while R-spondin 1 (*rspo1*) was significantly downregulated with 0.28-fold change. No significant changes were observed in other genes (Figure 5B). For the overexpression experiment, *hnrnpk* was overexpressed in CSTE cells, which was successfully confirmed by the extremely high expression level of *hnrnpk* gene examined by fluorescence microscopy (Figure 5C). In detail, the expression of *foxl2* and anti-Müllerian hormone (*amh*) was significantly downregulated (0.69-fold and 0.43-fold, respectively), while *cyp19a1a* and neuralized E3 ubiquitin protein ligase 3 (*neurl3*) were significantly upregulated (1.90-fold and 2.05-fold, respectively). The expression of other genes showed no significant changes (Figure 5D).

### 3.6. Transcriptomic Landscape Reveals Mechanisms of Hnrnpk-Induced Reprogramming in CSTE

**Figure 6:**
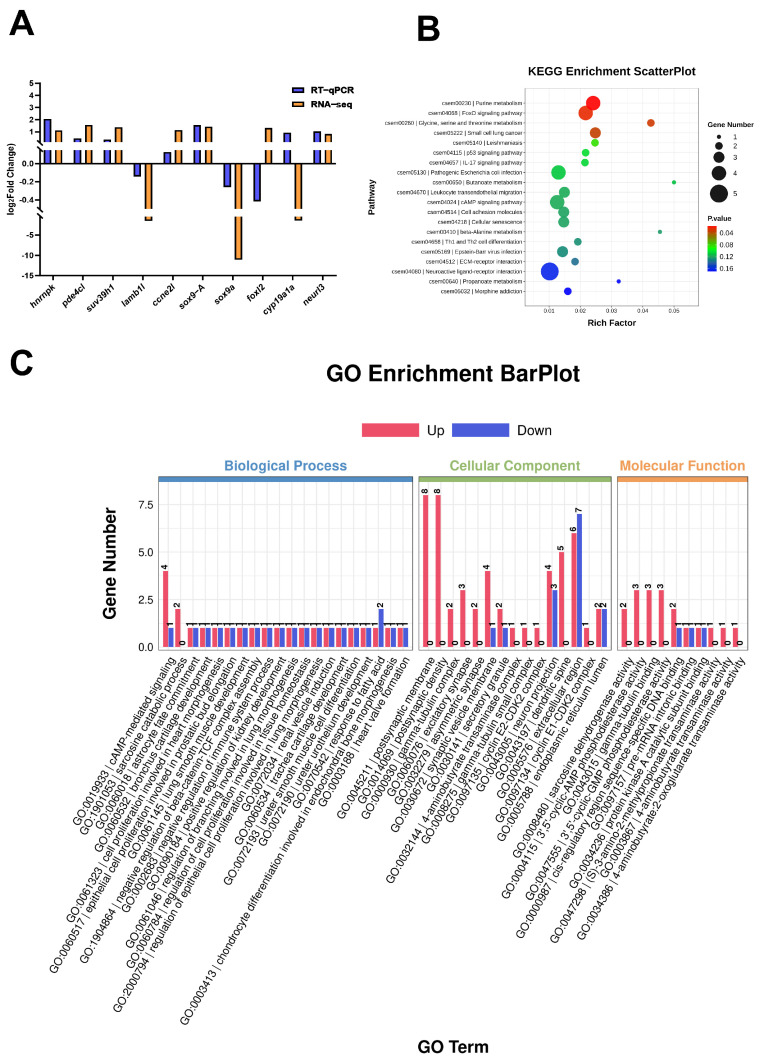
Transcriptomic landscape reveals mechanisms of *hnrnpk*-induced reprogramming in CSTE cells. (**A**) qPCR validation of differential genes. (**B**) KEGG pathway-enrichment analysis of DEGs (top 20 pathways). Bubble plot shows significantly enriched pathways. (**C**) GO functional enrichment analysis of DEGs. Significantly enriched terms in Biological Process (BP), Molecular Function (MF), and Cellular Component (CC) are shown.

To further elucidate the transcriptional regulation of *hnrnpk* in sex control and differentiation in *C. semilaevis*, the transcriptome sequencing analyses were subsequently performed in the *hnrnpk*-overexpression CSTE. A total of 126 significantly differentially expressed genes (DEGs) were identified compared to the control group (|log_2_FC| ≥ 1, *Q* < 0.05), comprising 89 up-regulated and 37 down-regulated genes. Among these, two *sox9* gene transcripts showed opposing expression patterns. Briefly, *sox-9-A* was significantly up-regulated (log_2_FC = 1.42), while *sox9a* was significantly down-regulated (log_2_FC = −11.05). In addition, several other sex-related genes were significantly altered. The cAMP-specific 3′,5′-cyclic phosphodiesterase 4C-like (*pde4cl)*, G1/S-specific cyclin-E2-like (*ccne2l*) and histone methyltransferase SUV39H1 (*suv39h1*) were significantly up-regulated with the fold change of log_2_FC = 1.57, log_2_FC = 1.15, and log_2_FC = 1.37, respectively. Conversely, the basement membrane component LAMB1-like (*lamb1l*) was significantly down-regulated (log_2_FC = −1.55) (Figure 6A).

KEGG pathway enrichment analysis showed that these DEGs were significantly enriched in pathways, among which the top 20 significantly enriched pathways were listed in Figure 6B. The results revealed that DEGs were mainly enriched in the FoxO-signaling pathway, glycine, serine, and threonine metabolism, and the small cell lung cancer pathways (Figure 6B).

GO functional enrichment analysis revealed profound cellular state transitions across three domains, including Biological Process (BP), Molecular Function (MF), and Cellular Component (CC) (Figure 6C). In BP, the DEGs were enriched in cAMP-mediated signaling, sarcosine catabolic process, astrocyte fate commitment, bronchus cartilage development, and so on. In MF, the DEGs were significantly enriched in terms, including sarcosine dehydrogenase activity, 3′,5′-cyclic-AMP phosphodiesterase activity, gamma-tubulin binding, and so on. In CC, significantly enriched terms included postsynaptic membrane, postsynaptic density, gamma–tubulin complex, and so on. In addition, several GO terms associated with the function of sex control and differentiation, such as male germ-line sex determination, Sertoli cell differentiation, male gonad development, etc., were also significantly enriched as shown in Table 3.

## 4. Discussion

*Hnrnpk* is a key member of the HNRNP family. *Hnrnpk* could be involved in the functions of transcriptional regulation, RNA splicing, translational control, DNA repair, and chromatin remodeling [3,4]. It also plays the key roles in spermatogenesis and female reproduction, as well as reproduction centers on precise post-transcriptional regulation [10,11]. Even though several investigations have been well-studied on *hnrnpk* in higher vertebrates, the function of *C. semilaevis hnrnpk* gene targeted by miRNAs in participating in regulating sex control and differentiation during the development of *C. semilaevis* was still limited. In this regard, miR-460-5p was predicted to target *hnrnpk*, which was verified by dual-luciferase reporter assay in HEK 293T cells, and the regulatory functions of *hnrnpk* in sex control and differentiation were explored following significantly different expression levels of *hnrnpk* in *C. semilaevis* gonadal tissues during different developmental stages, and the overexpression and knockdown analyses in the gonadal cells, based on the basic information of the identification of *hnrnpk*, including the full-length cDNA and protein sequences.

### 4.1. Structural Conservation and Female-Biased Expression of Hnrnpk

The deduced protein sequence of the cloned *C. semilaevis hnrnpk* contained highly conserved domains, showing structural conservation with those of other vertebrates, especially in teleosts. The gene structure of *C. semilaevis hnrnpk* has been determined to encode a 433-amino acid protein containing three canonical KH domains and a C-terminal ROKNT repeat region, which demonstrated a well conservation in the genomic organizations of other fish species, even higher vertebrates. Therefore, these findings revealed a high conservation of teleost *hnrnpk* in the evolution. The high phylogenetic and structural conservation of HNRNPK from teleosts to mammals suggests that its core functions are evolutionarily conserved in vertebrates [35].

To investigate the functions of *C. semilaevis hnrnpk*, tissue distribution analysis was performed, which revealed that *hnrnpk* was most abundantly expressed in the gonad, with females exhibiting consistently higher expression levels than males across all examined tissues. During gonadal development, expression levels in females peaked at the period of sexual maturity (1y) and still maintained a high level (2y), significantly exceeding those in males. This developmental stage coincides with the critical period of gonadal maturation [36,37], which suggested a female-biased expression pattern of *hnrnpk* in *C. semilaevis*. For example, in rats, *hnrnpk* was expressed in neonatal ovaries, and its knockdown disrupts primordial follicle assembly and increases oocyte apoptosis [11]. In the African clawed frog (*Xenopus laevis*), *hnrnpk* was present in oocytes, eggs, and early embryos, where it associates with maternal mRNAs [8]. Moreover, in Japanese flounder (*P. olivaceus*), a related KH domain-containing RNA-binding protein, *fmr1*, was highly expressed in the ovary [38], suggesting that KH domain proteins might play key roles in teleost ovarian development. In this study, the female-biased expression of *hnrnpk*, particularly its dynamic changes during gonadal development, strongly suggests that this gene may play a role in sex-related processes, providing a foundation for subsequent investigations into its upstream regulatory network and downstream functional mechanisms.

### 4.2. Transcriptional and Post-Transcriptional Regulation of Hnrnpk in C. semilaevis

To elucidate the molecular basis underlying the female-biased expression of *hnrnpk*, we systematically analyzed its upstream regulatory mechanisms. Promoter activity assays revealed that *hnrnpk* transcription is coordinately regulated by multiple sex-related transcription factors: *sox2* and *c-Jun* significantly enhanced its promoter activity, whereas *foxl2* and *ar* markedly suppressed it. It was reported that these factors functioned on sex-related bioprocesses in several previous studies. For instance, *sox2*, a pluripotency factor, played critical roles in oogenesis and early embryogenesis [39]. The factor *c-Jun* directly binded the promoters of *star* and other genes to regulate testicular function and steroidogenesis in teleosts [40]. *Foxl2*, a core transcription factor for ovarian differentiation and maintenance in teleosts, is specifically expressed in female granulosa cells [41,42], and *ar*, as the androgen receptor, mediates androgen signaling and spermatogenesis [43]. The coordinated regulation of the *hnrnpk* promoter by these sex-related factors showed an obvious antagonistic relationship between activators (*sox2*, *c-Jun*) and repressors (*foxl2*, *ar*), which provided a key transcriptional basis for its female-biased expression.

Post-transcriptional mechanisms also contribute to *hnrnpk* regulation. Several recent studies also indicated that *hnrnpk* genes could be regulated by different miRNAs to perform diverse functions. In this study, dual-luciferase reporter assays confirmed that miR-460a-5p directly targets the *hnrnpk* 3′UTR and significantly inhibits its expression. In mammals, accumulating evidence indicated that *hnrnpk* interacted with multiple miRNAs to regulate various biological processes. In porcine skeletal muscle satellite cells, miR-133a-3p directly targeted *hnrnpk*, establishing a regulatory axis that controled myogenic differentiation through modulation of *ucp2* expression [44]. In prostate cancer, miR-206 and miR-613 directly targeted the 3′UTR of *hnrnpk*, suppressing its expression and thereby inhibiting tumor cell proliferation and tumor growth [45]. In pediatric epilepsy, miR-873-5p directly targeted *hnrnpk*, contributing to oxidative stress and inflammatory responses [46].

However, which miRNAs regulate *hnrnpk* expression and what functions they mediate in fish remain unexplored. Notably, miR-460-5p responds to environmental temperature changes in birds [16], while the half-smooth tongue sole exhibits temperature-sensitive sex determination [2]. The miR-460a-5p-mediated inhibition of *hnrnpk*, potentially acting in concert with its differential expression between female and male gonads and integrating environmental signals, may collectively shape the sex-specific expression pattern of *hnrnpk*. However, the precise regulatory network needs to be investigated by deeper studies in the future.

### 4.3. Cell Type-Dependent Regulatory Effects of C. semilaevis hnrnpk on Sex-Related Genes in CSO and CSTE Cells

To investigate the role of *hnrnpk* in sex regulation, we performed loss-of-function and gain-of-function experiments in CSO and CSTE cells, respectively. In *hnrnpk*-knockdown CSO cells, several sex-related genes were significantly regulated. Briefly, female-related genes (*foxl2* and its downstream target *cyp19a1a*) were significantly upregulated. Loss-of-function studies have firmly established *hnrnpk* as a critical regulator of mammalian reproduction. In mice, germ cell-specific deletion of *hnrnpk* leads to spermatogenic arrest and complete male infertility [10,47]. In rats, siRNA-mediated knockdown of *hnrnpk* in neonatal ovaries disrupts primordial follicle assembly and increases oocyte apoptosis [11]. In vertebrates, the expression of *hnrnpk* in reproductive tissues has also been documented. In *X. laevis*, *hnrnpk* is present in oocytes, eggs, and early embryos, where it associates with maternal mRNAs [8]. In fish, however, direct evidence for *hnrnpk* expression or function in gonads is lacking, with most studies on *hnrnpk* family members focusing on antiviral immunity [6,48].

It was well known that *foxl2* played as a core transcription factor for ovarian differentiation in teleosts, which could directly activate *cyp19a1a* transcription [41,49], and *cyp19a1a* encodes aromatase, which catalyzes estrogen synthesis and serves as a key rate-limiting enzyme for female sex maintenance [50]. Loss-of-function studies have shown that disruption of either gene leads to female-to-male sex reversal or impaired ovarian development [51,52], underscoring their critical roles in establishing the female reproductive program. Consistently, *rspo1* is an activator of the WNT/β-catenin-signaling pathway and participates in ovarian differentiation in fish, with its expression levels closely associated with female development [53,54]. In *C. semilaevis*, *rspo1* has also been confirmed to exhibit female-biased expression and to be involved in the regulation of the WNT-signaling pathway [53]. In the present study, *rspo1* was significantly downregulated upon *hnrnpk* knockdown, suggesting that *hnrnpk* may be involved in the regulatory network of female-related genes in CSO cells, potentially through the modulation of the WNT-signaling pathway via *rspo1*. These findings may imply that the *C. semilaevis hnrnpk* gene can be used as a potential regulatory factor to mediate the sex differentiation or development of the ovary in *C. semilaevis*.

In *hnrnpk*-overexpression CSTE cells, a relatively more complete regulatory pattern of *C. semilaevis hnrnpk* gene was identified in sex control and regulation. In the current study, the expression of *foxl2* and *amh* was significantly suppressed, and *neurl3* was dramatically upregulated. Similarly, in mammals, the essential role of *hnrnpk* in male reproduction was demonstrated through both expression and functional studies. In mice, *hnrnpk* was highly expressed in the testis, localizing to the nuclei of pachytene spermatocytes, round spermatids, and Sertoli cells [55], with dynamic expression patterns also observed during testis development in rats and pigs [56]. Loss-of-function studies have further shown that germ cell-specific deletion of *hnrnpk* could cause spermatogenic arrest at the pachytene stage and complete male infertility [10,57]. Mechanistically, *hnrnpk* directly binded to the 3′UTR of piRNA pathway transcripts, enhancing their translational efficiency and thereby regulating piRNA production and spermatogenesis [10]. Collectively, these findings established *hnrnpk* as an indispensable regulator of male reproductive development in mammals. Unlike previous studies that primarily employed loss-of-function strategies (knockout or knockdown) to explore the reproductive function of *hnrnpk* in mice, the present study provided the first evidence in vertebrates that overexpression of *hnrnpk* also could modulate sex-related gene expression. *Amh*, a member of the TGF-β superfamily, serves as a key regulator of male gonadal development in teleosts [58,59]. In *C. semilaevis*, *amh* exhibits gonad-specific expression, with significantly higher expression levels in males than in females [60], and its downregulation disrupts the male maintenance program [59]. Moreover, *neurl3*, an E3 ubiquitin ligase located on the Z chromosome, regulates spermatogenesis in half-smooth tongue sole [61]. Consistent with its role in male reproduction, significantly downregulated amh and significantly upregulated *neurl3* were found upon *hnrnpk* overexpression in CSTE, further supporting the involvement of *hnrnpk* in male reproductive and spermatogenesis pathways.

Interestingly, the *cyp19a1a* gene was still significantly upregulated, which might also be due to a compensatory mechanism. Due to the suppressed expression of *foxl2*, *cyp19a1a*, as the downstream target gene of *foxl2,* might receive fewer signals from *foxl2* to stimulate its expression, and then, *foxl2*-independent alternative pathways might be activated to enhance the expression of *cyp19a1a*. These alternative pathways were also demonstrated by previous studies. The *cyp19a1a* transcriptional regulation in fish was complex, and multiple factors, including Nr5a1 (Sf1) and cAMP signaling, participated in its expression control [50,62]. Meanwhile, the male-determining gene *dmrt1* showed no significant change. *Dmrt1*, a Z chromosome-linked male-determining gene, is essential for testicular development [63]. The unchanged *dmrt1* expression indicates that the site where *C. semilaevis hnrnpk* performs its function might be located downstream of *dmrt1*, which means that *C. semilaevis hnrnpk* could participate in the regulation of gonadal differentiation and development, but not in the sex determination. However, more efforts are urgently needed to verify our hypothesis in the future.

These results indicated that *hnrnpk* expression should be maintained within a precise range in *C. semilaevis*. The deficiency of *hnrnpk* in the CSO upregulated the expression of *foxl2* and *cyp19a1a*, while its excess in the CSTE similarly induced abnormal *cyp19a1a* activation. This effect pattern might reveal the dosage sensitivity of *hnrnpk* function in *C. semilaevis*. Notably, *hnrnpk* exhibited context-dependent regulation of *cyp19a1a*. In the CSO, its upregulation accompanied *foxl2* upregulation, aligning with the classical pattern, but in the CSTE, its upregulation coexisted with *foxl2* suppression, suggesting *foxl2*-independent alternative pathways. However, this complex pattern prompted us to further explore the underlying mechanisms through transcriptome sequencing.

### 4.4. Transcriptomic Analyses Further Supported the Regulatory Functions of Hnrnpk in C. semilaevis Gonad Development

To further explore the mechanism underlying the paradoxical uncoupling of *foxl2* and *cyp19a1a* observed in *hnrnpk*-overexpressing CSTE cells—specifically, the upregulation of *cyp19a1a* despite *foxl2* suppression—we performed transcriptome sequencing on CSTE cells overexpressing *hnrnpk*. Transcriptome analysis revealed significant changes in multiple genes critical for sex determination and gonadal development. Most notably, the two isoforms of *sox9* exhibited completely opposite expression patterns. The canonical male isoform *sox9a* was dramatically suppressed (log_2_FC = −11.05), while a functionally uncharacterized non-canonical isoform, designated as *sox9-A*, was significantly upregulated (log_2_FC = 1.42). *Sox9* is central to the vertebrate sex determination cascade, driving Sertoli cell differentiation in mammals [64,65,66]. In teleosts, *sox9a* has been established as a critical regulator of testicular development, showing testis-specific high expression in species such as *C. semilaevis* and *Oryzias latipes* [1,67]. These results indicated that *hnrnpk* overexpression might directly disrupt the core program of male sex maintenance.

In addition, KEGG pathway-enrichment analysis revealed significant enrichment in several pathways associated with gonadal development. For instance, the FoxO-signaling pathway implicated in ovarian follicle development and oocyte maturation in both mammals and fish [68,69] was significantly enriched. S-phase kinase-associated protein 2 (*skp2*), a gene within this pathway, was significantly upregulated. *Skp2* was reported to promote cell cycle progression by targeting *p27Kip1* for degradation [70]. Enrichment of the FoxO pathway suggested that *hnrnpk* overexpression might aberrantly activate ovarian cycle progression, thereby promoting development programs of ovary in *C. semilaevis*. Moreover, the purine metabolism pathway, closely linked to cAMP signaling [71], was also significantly enriched. It was reported that the cAMP levels were regulated by PDE4 family members to influence Sertoli cell responsiveness to FSH (Follicle-Stimulating Hormone) during spermatogenesis [72], and cAMP also served as a critical second messenger, enabling Sertoli cells to respond to FSH [73]. In this study, multiple cAMP-specific phosphodiesterase genes within this pathway, including cAMP-specific 3′,5′-cyclic phosphodiesterase 4B-like (*pde4bl*) and cAMP-specific 3′,5′-cyclic phosphodiesterase 4C-like (*pde4cl*), were significantly upregulated. The marked upregulation of PDEs indicated the suppression of cAMP signaling, which might further compromise the male maintenance program.

## 5. Conclusions

**Figure 7:**
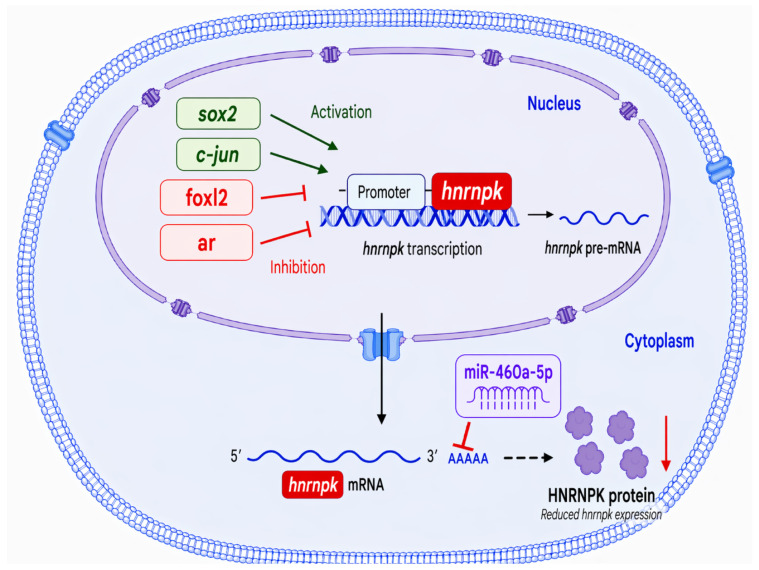
A hypothesis of the potential regulatory mechanism of the *hnrnpk* gene targeted by miR-460a-5p in the gonadal differentiation and development of *C. semilaevis*.

In this study, we characterized the role of *hnrnpk* in regulating sex-related gene expression in *C. semilaevis*. The CDS of *hnrnpk* was cloned and identified, which was used to reveal its female-biased expression during gonadal development. As shown in Figure 7, our findings also demonstrated that *hnrnpk* expression could be regulated at both transcriptional (*sox2*/*c-Jun* activation, *foxl2*/*ar* repression) and post-transcriptional (miR-460a-5p targeting) levels. Meanwhile, functional assays showed that *hnrnpk* could regulate *cyp19a1a* in a cell type-dependent and dose-sensitive manner. Transcriptome analyses further demonstrated that *hnrnpk* overexpression could suppress the canonical male isoform *sox9a* and activate multiple sexual regulation-related signaling pathways. Overall, our findings demonstrate that *hnrnpk*, a female-biased gene regulated by multiple mechanisms, plays multiple roles in gonadal development through miR-460a-5p targeting and *sox9* isoform switching. This study will offer new insights into sex determination for *C. semilaevis hnrnpk* and also provide a potential target for monosex breeding in aquaculture.

## Figures and Tables

**Table 1:** Primers used in present study.

Primers	Sequence (5′—3′)	Primer Application
*hnrnpk*-CDS-F *hnrnpk*-CDS-R *hnrnpk*-F *hnrnpk*-R *β-actin*-F *β-actin*-R *hnrnpk*-pro-F *hnrnpk*-pro-R miRNA-460a-5p-s miRNA-460a-5p-a siRNA-1-s siRNA-1-a siRNA-2-s siRNA-2-a siRNA-3-s siRNA-3-a pcDNA3.1-*hnrnpk*-F pcDNA3.1-*hnrnpk*-R *foxl2*-F *foxl2*-R *cyp19a1a*-F *cyp19a1a*-R *neurl3*-F *neurl3*-R *dmrt1*-F *dmrt1*-R *amh*-F *amh*-R *wnt4*-F *wnt4*-R *ctnnb1*-F *ctnnb1*-R *figla*-F *figla*-R *rspo1*-F *rspo1*-R *sox9*-F *sox9*-R *pde4cl*-F *pde4cl*-R *suv39h1*-F *suv39h1*-R *lamb1l*-F *lamb1l*-R *ccne2l*-F *ccne2l*-R *sox9-A*-F *sox9-A*-R *sox9a*-F *sox9a*-R	ATGGAGACAGAAATTGAA CAGCAAATGACCAGAGTA GATGGTTGAGCTTCGCAT GGGACTGACACACTGGCA TTCCAGCCTTCCTTCCTT TACCTCCAGACAGCACAG ATCTGCGATCTAAGTAAGCTTAGGCAAACGGAACCTGGATAT CAGTACCGGAATGCCAAGCTTGGTCTTCTGACAGTCAAAGCGAC CCUGCAUUGUACACACUGUGUG CACAGUGUGUACAAUGCAGGUU GGAUGCAGAUGAACAGAAA/dT//dT/ UUUCUGUUCAUCUGCAUCC/dT//dT/ CGAGGAAUUCAGACGAGAU/dT//dT/ AUCUCGUCUGAAUUCCUCG/dT//dT/ GAAGAGUACCAGCAGUAUA/dT//dT/ UAUACUGCUGGUACUCUUC/dT//dT/ GCTAGCGTTTAAACTTAAGCTTATGGAGACAGAAATTGAACAGC AGGATCCCATTGTACCAAGCTTCAGCAAATGACCAGAGTACTG CCGGCCTGTGAAGAC TGCAGGTACTTAGGCG GGTGAGGATGTGACCCAGTGT ACGGGCTGAAATCGCAAG CTGGTGTTTAGCAGCCGTCCT CCAGAACTCCAGCACTGACCC GGAGGAAGAACTTGGGATTTG AGGTAGGAGGTTGCTGGG CAGCACAAACCAGGGAAG AACACCAGGAGCAGGACA ACATGTGAGCGGTTACGAGG CACTTTGCCAAACACAGGCA TTTGTGCCCTACGTCACCTC ATGAGTGGCCAGTGTGATGG AGGAAGCCCAGTAAAGTA TTAGGAAATCAGACCCAC ATCAAGTGTAAGCCCAAG TTCCTCATTCCAAAGTAT GTCCGTTTGTAGAGGAGGCA GCCCATACTGGACGCAG AGGCGGAGTCATCGGGCT CGGAAGAGTTGTGGGGCG CCAGACTCACACTCATCC TCTTACACTCACATCCCA TCACAGCAACATCAGCCT TGTCCCGTTACATCCATC TCAGGAGCAGAAGCACAA GACTCGACACCAGCCAAC TGAAGATGACGGAGGAAC CTCAGGATTCCGTTCTCG ACAGCCATGCTGGATTGC TGGGCCCCAACATTAGCT	CDS cloning CDS cloning qPCR qPCR qPCR qPCR promoter cloning promoter cloning miRNA miRNA siRNA siRNA siRNA siRNA siRNA siRNA overexpression overexpression qPCR qPCR qPCR qPCR qPCR qPCR qPCR qPCR qPCR qPCR qPCR qPCR qPCR qPCR qPCR qPCR qPCR qPCR qPCR qPCR qPCR qPCR qPCR qPCR qPCR qPCR qPCR qPCR qPCR qPCR qPCR qPCR

**Table 2:** Characteristics of the CDS region and its deduced protein of *hnrnpk* in *C. semilaevis*.

Parameter	Value
CDS length	1302 bp
Number of amino acids	433 aa
Predicted molecular weight	48.16 kDa
theoretical isoelectric point	6.74

**Table 3:** Significantly enriched Biological Process GO terms associated with sex control and differentiation.

GO_ID	GO Term	Gene Count	*p* Value	Q. Value	Up-Regulated Genes	Down-Regulated Genes	ZScore
GO:0019100	Male germ-line sex determination	2.0	0.00009	0.004	transcription factor Sox-9-A	SRY (sex determining region Y)-box 9 isoform X1	−0.577
GO:2000020	positive regulation of male gonad development	2.0	0.00019	0.007	transcription factor Sox-9-A	SRY (sex determining region Y)-box 9 isoform X1	0.000
GO:0060008	Sertoli cell differentiation	2.0	0.00031	0.009	transcription factor Sox-9-A	SRY (sex determining region Y)-box 9 isoform X1	0.000
GO:0060009	Sertoli cell development	2.0	0.00046	0.011	transcription factor Sox-9-A	SRY (sex determining region Y)-box 9 isoform X1	0.816
GO:0030238	male sex determination	1.0	0.02778	0.098	transcription factor Sox-9-A		1.000
GO:0008584	male gonad development	3.0	0.00967	0.065	transcription factor Sox-9-A, tumor necrosis factor ligand superfamily member 10-like	SRY (sex determining region Y)-box 9 isoform X1	2.749

## Data Availability

Transcriptome data were deposited in the SRA database under BioProject accession PRJNA1438903 and SRA study accession SRP684648.

## Supplementary Materials

The following supporting information can be downloaded at: https://www.mdpi.com/article/10.3390/ani16091327/s1, Figure S1: The CDS of hnrnpk in C. semilaevis, total length 1302 bp. Figure S2: Multiple sequence alignment of HNRNPK amino acid sequences. Multiple sequence alignment of HNRNPK homologous protein sequences from 17 species was performed using MEGA software with the ClustalW algorithm. The alignment results were visualized using ESPript 3.0. Red background indicates strictly conserved residues across all aligned sequences, red characters denote conservative substitutions, and other colors represent regions with lower sequence conservation. Table S1: Biological Process; Molecular Function; Cellular Component.
