# Supplementary material for: The Heterogeneous Nuclear Ribonucleoprotein K (hnrnpk) Gene Targeted by miR-460a-5p Functions in the Gonadal Differentiation and Development in Chinese Tongue Sole (Cynoglossus semilaevis)
Source: Animals (Basel). 2026 Apr 27;16(9):1327. doi: 10.3390/ani16091327 (PMC13162840; doi:10.3390/ani16091327)
Supplement: Supplementary file 1 [file animals-16-01327-s001.zip › animals-4249555-supplementary.pdf]

Supplementary Materials: CDS sequence of *hnrnpk* in in Chinese tongue sole (*Cynoglossus semilaevis*) and results of multiple sequence alignment

Manuscript title: The heterogeneous nuclear ribonucleoprotein K (*hnrnpk*) gene targeted by miR-460a-5p functions in the gonadal differentiation and development in Chinese tongue sole (*Cynoglossus semilaevis*)

1. CDS sequence of *hnrnpk* in *C. semilaevis*

```
ATGGAGACAGAAATTGAACAGCACGAAGAATCATTTAGCAACAACGAGACAAACGGTAAG
 M  E  T  E  I  E  Q  H  E  E  S  F  S  N  N  E  T  N  G  K
CGCCCTGCTGAGGATGCAGATGAACAGAAATCGTTCAAGCGCTCGAGGAATTCAGACGAG
 R  P  A  E  D  A  D  E  Q  K  S  F  K  R  S  R  N  S  D  E
ATGGTTGAGCTTCGCATCCTCCTGCAGAGCAAAAACGCAGGAGCTGTGATTGGAAAGGGT
 M  V  E  L  R  I  L  L  Q  S  K  N  A  G  A  V  I  G  K  G
GGTAAAAACATCAAAGCCCTGCGTACAGACTACAATGCCAGTGTGTCAGTCCCAGACAGC
 G  K  N  I  K  A  L  R  T  D  Y  N  A  S  V  S  V  P  D  S
AGTGGGCCTGAGCGCATCCTGAGCATCAGTGCTGATATTGAGACAGTTGGAGAAATCCTG
 S  G  P  E  R  I  L  S  I  S  A  D  I  E  T  V  G  E  I  L
CTCAAGATTATTCCAACACTTGAAGAGTACCAGCAGTATAATGGTATGGATTTTGACTGT
 L  K  I  I  P  T  L  E  E  Y  Q  Q  Y  N  G  M  D  F  D  C
GAGCTGCGTTTGCTGATCCATCAGAGCCTTGCGGGCTCAATCATTGGGGTGAAGGGAGCC
 E  L  R  L  L  I  H  Q  S  L  A  G  S  I  I  G  V  K  G  A
AAAATTAAGGAGCTCCGGGAGAATACAAAAACCAGCATCAAGCTGTTTCAGGAGTGCTGT
 K  I  K  E  L  R  E  N  T  K  T  S  I  K  L  F  Q  E  C  C
CCTCAGTCAACAGACCGTGTGGTGCTAGTTGGTGGTAAAACAGAGAGAGTGGTGGAGTGT
 P  Q  S  T  D  R  V  V  L  V  G  G  K  T  E  R  V  V  E  C
ATCAAGACCATGCTTGAGCTCATCGCTGATGCCCCCATAAAAGGTCGCGCCCAGCCCTA
 I  K  T  M  L  E  L  I  A  D  A  P  I  K  G  R  A  Q  P  Y
GACCCAAACTTCTACGACGAAACCTATGAATACGGTGGATTCACCATGATGTTTGAAGAC
 D  P  N  F  Y  D  E  T  Y  E  Y  G  G  F  T  M  M  F  E  D
CGGGGCAGCAGCCGAAGAATGATGGGAGGCTTCCCAATGCGTGGTGGAAGGTCCAGCGCT
 R  G  S  S  R  R  M  M  G  G  F  P  M  R  G  G  R  S  S  A
GGAGACCGTGGCTATGAAAGGATGTCTTCCAGCAGAGGCCCACGTGGTCCCATGCCCCCC
 G  D  R  G  Y  E  R  M  S  S  S  R  G  P  R  G  P  M  P  P
TCTCGTCGGGACTACGATGATATGAGCCCCCGTCGAGGTCCTCCTCCTCACCCAAGCAGA
 S  R  R  D  Y  D  D  M  S  P  R  R  G  P  P  P  H  P  S  R
GTCAGCAGGGGAAGTAGCCGTGGACGCAACATGTCCATTGGACATCCACACAGAGGAGGA
 V  S  R  G  S  S  R  G  R  N  M  S  I  G  H  P  H  R  G  G
GATGATCGTTACTATGACTCGTACCGTGGCTCAGATGAAAGGTCAAATGACAGAAGAAGC
 D  D  R  Y  Y  D  S  Y  R  G  S  D  E  R  S  N  D  R  R  S
AGACCAGATCGCTACAGCGATAGCATGGGGGGGGCATATGACAACAGTTCATCTTGGGAT
 R  P  D  R  Y  S  D  S  M  G  G  A  Y  D  N  S  S  S  W  D
AGCTACCAGTCAGGTGGACGAGGCTCCTACAGTGATATAAGTGGTCCTACTGTCACCACA
 S  Y  Q  S  G  G  R  G  S  Y  S  D  I  S  G  P  T  V  T  T
CAAGTGACGATCCCCAAAGATCTTGCTGGGTCTATTATTGGTAAGGGAGGCCAGAGGATC
 Q  V  T  I  P  K  D  L  A  G  S  I  I  G  K  G  G  Q  R  I
AAACAGATCCGTCATGACTCTGGAGCCTCTATCAAAATCGATGAGCCTCTGGAAGGTGCT
 K  Q  I  R  H  D  S  G  A  S  I  K  I  D  E  P  L  E  G  A
GAGGATCGAATAATCACCATTATTGGCACACAGGATCAAATCCAGAATGCTCAGTACCTT
 E  D  R  I  I  T  I  I  G  T  Q  D  Q  I  Q  N  A  Q  Y  L
CTACAGCACAGTGTGAAGCAGTACTCTGGTCATTTGCTGTAG
 L  Q  H  S  V  K  Q  Y  S  G  H  L  L  *
```

Supplementary Materials Figure S1: The CDS of *hnrnpk* in *C. semilaevis*, total length 1302 bp.

2. Multiple sequence alignment of *hnrnpk* CDS sequences from different species.

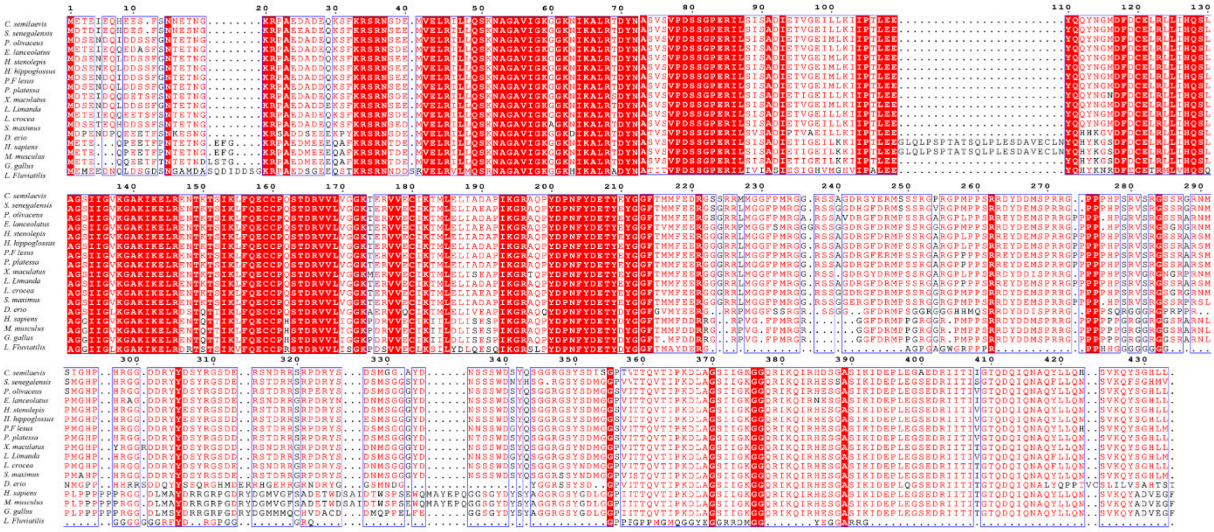

Supplementary Materials Figure S2: Multiple sequence alignment of HNRNPK amino acid sequences. Multiple sequence alignment of HNRNPK homologous protein sequences from 17 species was performed using MEGA software with the ClustalW algorithm. The alignment results were visualized using ESPript 3.0. Red background indicates strictly conserved residues across all aligned sequences, red characters denote conservative substitutions, and other colors represent regions with lower sequence conservation.
